# A novel hybrid approach to treatment of multiple mesenteric aneurysms in a patient with celiac artery occlusion from Suspected chronic median arcuate ligament syndrome

**DOI:** 10.1016/j.jvscit.2025.101730

**Published:** 2025-01-15

**Authors:** Prashanth S. Iyer, Vamsi K. Potluri, Jennifer L. Worsham, Christine L. Shokrzadeh, Zulfiqar Cheema, Charlie Cheng, Michael B. Silva

**Affiliations:** a Division of Vascular Surgery, Department of Surgery, University of Oklahoma, Tulsa, OK; b Division of Vascular Surgery, Department of Surgery, Loyola University Medical Center, Hines, IL; c Division of Vascular Surgery, Department of Surgery, University of Texas Medical Branch, Houston, TX; d Clear Lake Specialties, Houston, TX; e Houston Methodist Hospital, Department of Cardiovascular Surgery, Houston, TX

**Keywords:** Aneurysms, Median arcuate ligament syndrome

## Abstract

The management of visceral artery aneurysms is evolving with endovascular coiling and covered stent placement used as alternatives to open repair. Celiac artery occlusion or compression complicates ablative endovascular management. The purpose of this report was to discuss the etiology of this uncommon phenomenon and describe a novel hybrid approach to treatment.

Splanchnic artery aneurysms, defined as aneurysms of the celiac trunk, superior mesenteric artery, inferior mesenteric artery, and their respective branches, are rare and potentially life threatening. The incidence of splanchnic artery aneurysms is between 0.1% and 2.0% of all aneurysms.^1^, ^2^, ^3^, ^4^ Aneurysms of the pancreaticoduodenal arcade and the gastroduodenal artery are more likely to rupture and warrant an aggressive approach to treatment. Owing to the rarity of this condition, there is no consensus in the literature about precise indications for the repair of splanchnic artery aneurysms or the best type of surgical treatment.^2^^,^^5^ We describe the successful treatment of a patient with celiac occlusion and multiple aneurysms of the gastroduodenal and pancreaticoduodenal arteries using a hybrid approach with open aorto-hepatic artery bypass followed by intra-operative endovascular transcatheter coil embolization of the mesenteric artery aneurysms.

## Case report

Informed consent was obtained from this patient before publishing this report. A 50-year-old woman with a past medical history of hypertension, hyperlipidemia, and a 10-year smoking history presented with three days of worsening epigastric pain. She was found to have an elevated lipase and was initially treated for acute pancreatitis. A computed tomography (CT) scan of the abdomen showed a nonatherosclerotic celiac occlusion with an element of extrinsic compression and five mesenteric aneurysms, with the largest aneurysm measuring 4.6 cm off the gastroduodenal artery. Additionally, the patient mentioned a remote history of postprandial abdominal discomfort and weight loss secondary to this recurring pain. Although the patient did not have a diagnosis of median arcuate ligament syndrome, the CT scan findings combined with this history is consistent with symptomatic median arcuate ligament syndrome, although this diagnosis cannot be confirmed retrospectively. She had no history of stroke or transient ischemic attack, myocardial infarction, claudication, or any other symptomatic evidence of atherosclerotic disease. There is no family history of aneurysms.

**Fig 1:**
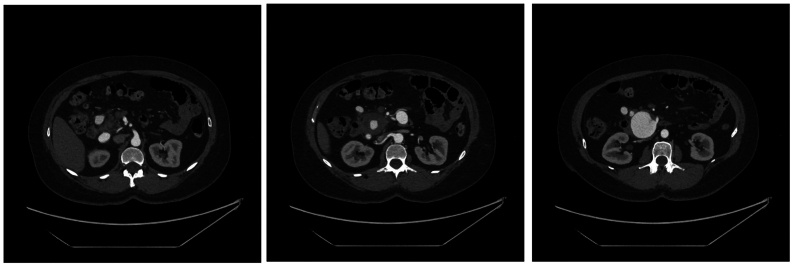
Axial cross sections of abdominal computed tomography (CT) scan with contrast shows multiple mesenteric aneurysms involving branches of the superior mesenteric artery, gastroduodenal artery, and pancreaticoduodenal arcade.

**Fig 2:**
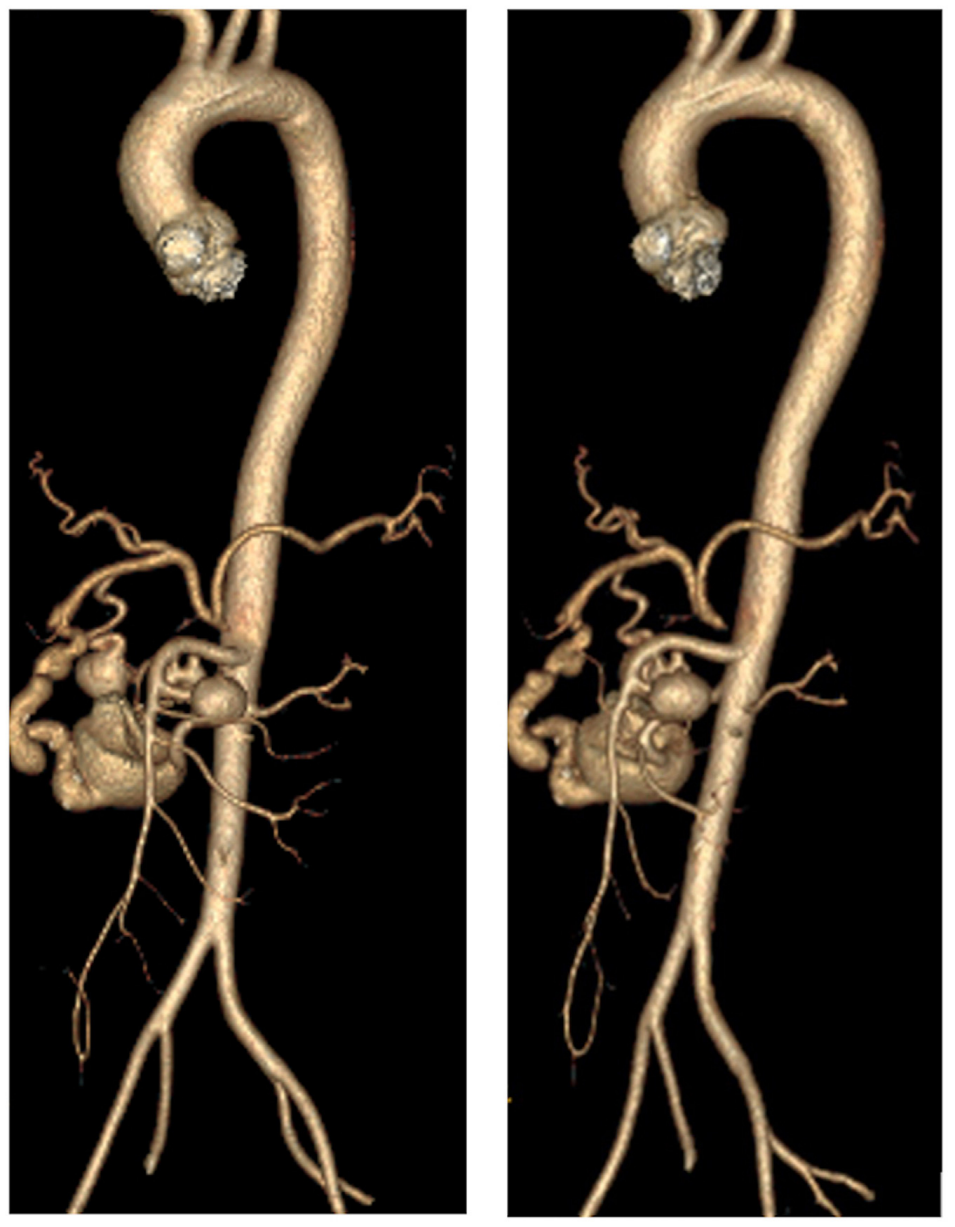
Three-dimensional reconstruction of computed tomography (CT) angiography demonstrating one aneurysm of the gastroduodenal artery (4.6 cm), two of the superior mesenteric artery (1.7 cm, <1.0 cm), and two of pancreaticoduodenal arcades (1.3 cm, 1.5 cm) with concurrent celiac occlusion.

CT angiography of the thorax, abdomen, and pelvis with three-dimensional reconstruction was then obtained to further identify the anatomy of her mesenteric aneurysms (Figs 1 and 2).

To rule out a mycotic aneurysm, a tagged white blood cell scan was completed with no remarkable findings. Typically, CT angiography is preferred over a white blood cell scan to diagnose a mycotic aneurysm owing to the variable sensitivity of the latter.^6^ However, in this case, the patient's recent episode of pancreatitis made it difficult to discern the etiology of the inflammation. A mesenteric arteriogram was obtained to better characterize the patient's anatomy and aneurysmal disease for operative planning. The angiogram demonstrated that the hepatic artery was perfused through collateralization from the superior mesenteric artery and retrograde flow through the gastroduodenal artery. Most of the patient's mesenteric aneurysms were in this pathway.

Given the patient's overall clinical presentation and number of aneurysms, we elected a surgical intervention. Although the patient was having an active episode of acute pancreatitis, making open surgery difficult, we believed the aneurysms were a contributing factor to the patient's abdominal pain and they could be the cause of her persistent pancreatic leak owing to compression and inflammation. Furthermore, given the size of the aneurysms—almost 5 cm in some places—rupture was a consideration. Finally, consideration was given to excluding each aneurysm; however, anatomical elimination of all collateral branches from the seven aneurysms risked devascularizing significant portions of circulation to the bowel.

**Fig 3:**
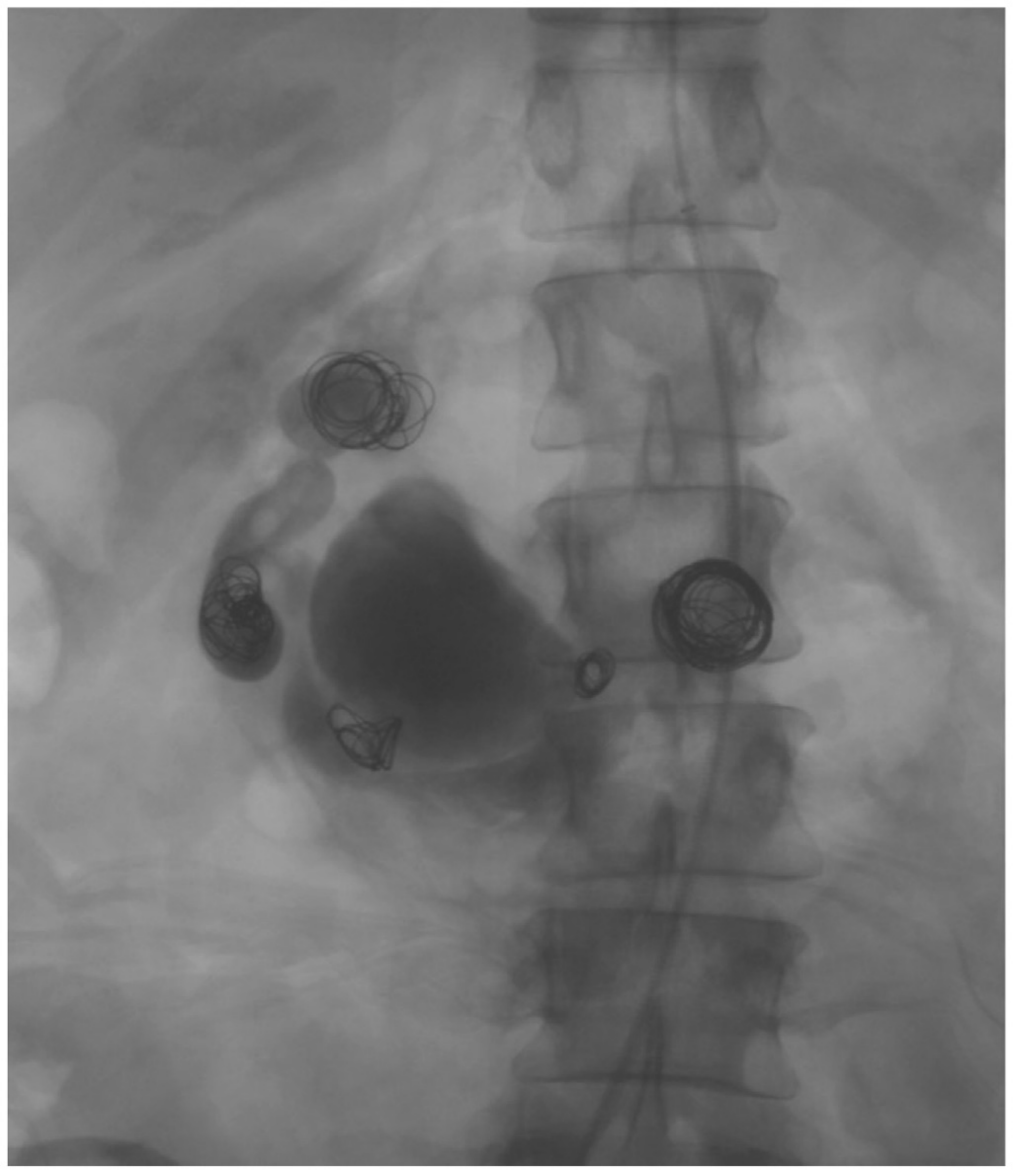
Pos-intervention stagnation of contrast through all mesenteric aneurysms.

**Fig 4:**
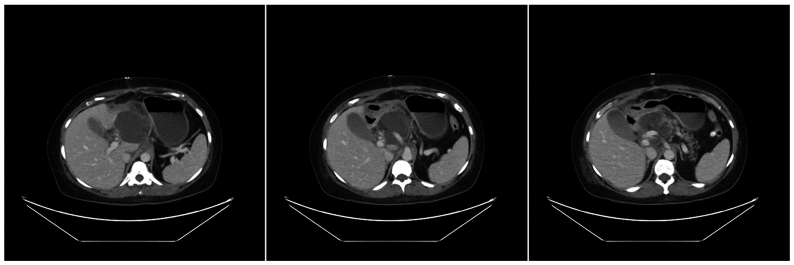
Axial cross sections of postoperative abdominal computed tomography (CT) scan with contrast shows peripancreatic fluid collection, involving portion of aortohepatic bypass graft.

An endovascular approach was attempted to stent the celiac artery and maintain splanchnic circulation during possible transcatheter coil embolization of the aneurysms. However, transfemoral and transbrachial attempts to recanalize the celiac occlusion were unsuccessful; there was no residual orifice for insertion of the endovascular device owing to a flush occlusion, so the procedure was converted to an open surgical repair. We proceeded to create an aortohepatic bypass between the supraceliac aorta and the common hepatic artery before ligation of the gastroduodenal artery. Although a vein would have been an ideal conduit for the bypass, the patient had no suitable vein, so a polytetrafluorethylene graft was chosen for its material characteristics and expedience. We then took an endovascular approach to treatment by gaining arterial access via the ligated gastroduodenal artery and occluded the aneurysm sacs using a total of 9 interlocking coils (Boston Scientific, Marlborough, MA). A GELFOAM (Pharmacia and Upjohn Co, Kalamazoo, MI). Thrombin slurry was also injected into the largest aneurysm (4.6 cm) to further occlude blood flow. A postoperative angiogram demonstrated flow through the patent aortohepatic bypass graft with preservation of splanchnic circulation and occlusion of the aneurysms (Fig 3). Given the emergent nature of the surgery, the postoperative period was unsurprisingly complicated by worsening of her pancreatitis. She was found to have a peripancreatic fluid collection on a postoperative CT scan (Fig 4), into which a drain was placed by interventional radiology. The fluid was infected with *Serratia marcescens* and had high levels of amylase and lipase; it was thought to be a pancreatic abscess secondary to her severe episode of acute postoperative pancreatitis. She was discharged on levofloxacin with the drain in place, and the drain was later removed in clinic with a repeat CT scan showing complete resolution of the peripancreatic fluid collection. This initial postoperative course was followed by return of appetite and 2 years of weight gain necessitating laparoscopic gastric sleeve surgery, which occurred without complication and resulted in appropriate weight loss. She is now 5 years out from initial surgery and remains asymptomatic. Her graft remains patent by mesenteric duplex and CT scan.

## Discussion

Visceral aneurysms are rare but can be potentially fatal if they rupture. Unfortunately, given their rare nature, data on how to diagnosis and manage these aneurysms is scarce and primarily based on case reports.^7^ For gastroduodenal and pancreoduodenal aneurysms, the Society for Vascular Surgery strongly recommends intervening on all aneurysms regardless of size. The preferred intervention is coil embolization with liquid embolic agents.^8^ If coil embolization is not possible, the aneurysms should be treated with a covered stent or stent assisted coil embolization. If the patient has a suitable anatomy, flow-diverting, multilayer stents are an option, although they have not been well-studied.^8^ If flow cannot be maintained with an endovascular option, open surgical reconstruction is recommended.^8^ Finally, in patients with concomitant stenosis or occlusion, celiac artery reconstruction is recommended.^8^

The association between celiac artery stenosis or occlusion and aneurysms of the gastroduodenal and pancreaticoduodenal arteries has previously been reported in the literature. Corey et al^5^ reports that 24 of 36 aneurysms (66%) of these arteries were associated with severe celiac artery stenosis. Also, in a separate study de Perrot et al,^9^ five of six patients (83%) with aneurysms in these arteries have concurrent celiac artery stenosis or occlusion. The proposed pathophysiology behind this phenomenon is secondary to increased blood flow through these thin-walled arteries, because they serve as collateral circulation to perfuse organs typically supplied by the celiac trunk.^5^^,^^9^, ^10^, ^11^

Although endovascular therapy is becoming a more attractive option as it is further refined, data are limited on its efficacy compared with open bypass.^12^ A study conducted by Sharafuddin et al^13^ on 27 patients with total occlusions of mesenteric arteries found that 80% of patients were free of ischemic symptoms after 1 year using endovascular stenting and angioplasty and concluded that endovascular recanalization of mesenteric artery occlusion is both feasible and successful. Only 3 of these 27 patients suffered from celiac artery stenosis. In contrast, Ahanchi et al^12^ conducted a study on 121 patients to determine the efficacy of stenting of mesenteric arteries and found that only 18% of patients with celiac artery stenosis had primary patency after one year after treatment using balloon expandable stents, whereas freedom from bypass was 93% after 4 years. They concluded that celiac artery stenosis does not respond well to endovascular stenting. Their hypothesis was that the decrease in primary patency observed with celiac artery stenting was potentially influenced by poor anatomic location and the dynamic forces of respiration combined with the fixed ligament attachments of the diaphragm.

The management of visceral artery aneurysms is evolving with endovascular coiling and covered stent placement used as alternatives to open repair. Although open surgical treatment of digestive artery aneurysms offers good long-term results in terms of patency,^14^ postoperative risk of complication remains significant; Ghariani et al^2^ found that postoperative complications from open surgical repair were mainly respiratory (18%), digestive (18%), and renal (13%). Fankhauser et al^15^ reported on 176 patients with endovascular treatment of visceral artery aneurysms and found that initial intervention was successful in 98% of patients, with no complications from any percutaneous procedure; they concluded that minimally invasive management of visceral artery aneurysms, including coil embolization and endoluminal GELFOAM (Pharmacia and Upjohn Co) thrombin injection an can be used as effective treatment with a high success rate and low recurrence and morbidity.

## Conclusions

Aneurysms of the visceral arcade connecting the superior mesenteric and celiac artery circulation may be associated with increased flow from dynamic changes associated with celiac artery compression or occlusion. Considering these recommendations and studies, we present a safe but durable solution with our hybrid approach to management.

## Funding

None.

## Disclosures

None.
